# Explaining COVID-19 outbreaks with reactive SEIRD models

**DOI:** 10.1038/s41598-021-97260-0

**Published:** 2021-09-09

**Authors:** Kunal Menda, Lucas Laird, Mykel J. Kochenderfer, Rajmonda S. Caceres

**Affiliations:** 1 Department of Aeronautics & Astronautics, Stanford University, Stanford, CA USA; 2 MIT Lincoln Laboratory, Lexington, MA USA

**Keywords:** Epidemiology, Statistics, Computational science

## Abstract

COVID-19 epidemics have varied dramatically in nature across the United States, where some counties have clear peaks in infections, and others have had a multitude of unpredictable and non-distinct peaks. Our lack of understanding of how the pandemic has evolved leads to increasing errors in our ability to predict the spread of the disease. This work seeks to explain this diversity in epidemic progressions by considering an extension to the compartmental SEIRD model. The model we propose uses a neural network to predict the infection rate as a function of both time and the disease’s prevalence. We provide a methodology for fitting this model to available county-level data describing aggregate cases and deaths. Our method uses Expectation-Maximization to overcome the challenge of partial observability, due to the fact that the system’s state is only partially reflected in available data. We fit a single model to data from multiple counties in the United States exhibiting different behavior. By simulating the model, we show that it can exhibit both single peak and multi-peak behavior, reproducing behavior observed in counties both in and out of the training set. We then compare the error of simulations from our model with a standard SEIRD model, and show that ours substantially reduces errors. We also use simulated data to compare our methodology for handling partial observability with a standard approach, showing that ours is significantly better at estimating the values of unobserved quantities.

## Introduction

Having an accurate understanding of the spread of COVID-19 is essential to containing the virus effectively, and necessary for the deployment and allocation of resources. In order to understand why the spread appears to differ between communities, we require a mathematical model of disease spread capable of expressing the differences. A standard model of disease spread is the SEIRD model, in which each individual is either susceptible (S), exposed (E), infected (I), recovered (R), or dead (D)^1,2^. *Compartmental* SEIRD models consider only the aggregate number of individuals with each disease state, and specify a set of differential equations that govern how the compartmental populations change with time.

The well-studied compartmental SEIRD model is popular because of its simplicity^2^. This standard model, however, only predicts a single, clear peak in infections. While certain counties in the northeast of the United States appeared to exhibit this behavior for a while, most other counties do not.

In this work, we seek to extend the model and fit it to available data that can account for the diversity in COVID-19 outbreaks across the US. Specifically, we seek to learn a model that predicts clear peaks for counties in the northeast that had them, and predicts multiple, flatter peaks for those that did not.

In order to build models capable of expressing more realistic behavior, we relax two assumptions made by the standard compartmental SEIRD model:

1. Stationarity—the disease parameters remain constant over time, and,
2. Non-reactivity—the disease parameters remain constant regardless of the prevalence, i.e., the fraction of the population infected by the disease.

We hypothesize that relaxing these assumptions allows us to better explain the diversity of behavior seen in reality. Specifically, allowing disease parameters to vary with time allows us to account for implicit dependencies on virus mutations, weather, and changing cultural norms. Allowing them to depend on prevalence allows us to account for the reactive nature of a population’s behavior to the perceived threat of the virus, and heterogeneous patterns of interactions within populations in different geographical areas, which reflect in the peak prevalence levels reached.

To relax these assumptions, we consider a “reactive” compartmental SEIRD model (R-SEIRD) in which the transmission rate is a function of both time and the number of infected individuals. To avoid imposing an incorrect prior on the functional form of this relationship, we use a neural network to model it.

A common difficulty in fitting such models to available data is that of *partial observability*. SEIRD models have *states* that vary with time according to their dynamic parameters. Because the available data are not time-series of the models’ states, but only partial observations of it (daily new cases and deaths), we employ tools from system-identification^3^ in order to fit these models. Specifically, we use a technique called Certainty-Equivalent Expectation-Maximization (CE-EM)^4^, which was recently shown to be a reliable and low-variance method for learning the parameters of partially observable dynamical systems. A key benefit of this methodology is that we do not assume knowledge of the initial state of the population, an assumption typically made by related approaches^5–8^.

In this work, we present a methodology for fitting R-SEIRD models to available data, and validate our hypotheses by showing that learned models can explain the diversity of behavior seen across the United States, and that is more robust to partial observability. Through our experiments, we show that:

1. Learned R-SEIRD models produce behavior consistent with what was observed in counties across the United States,
2. The simulation error of R-SEIRD models is lower than that of standard SEIRD models when compared against trajectories from outbreaks across the United States, and,
3. CE-EM is better than standard methodologies for fitting SEIRD models to partially observed data.

The contributions of this work are, therefore, to:

1. Propose a novel model that relaxes the assumptions of the standard SEIRD model,
2. Provide a methodology that is novel for fitting such a model to available data, and,
3. Experimentally demonstrate that this model is capable of expressing the diversity of behavior observed across the United States, and that our methodology outperforms standard approaches in partially observed settings.

This paper is organized as follows. In the “Background” section, we formalize compartmental SEIRD models and review the CE-EM algorithm, as well as related approaches to fitting such models to data. In the “Methodology” section, we introduce the R-SEIRD model and describe our methodology for fitting it to available data. In the “Fitting COVID-19 epidemics across The United States” section, we fit the model to data from a selection of representative counties across the United States and show that it is able to reflect the observed behavior. In the “Inferring versus Assuming Initial Conditions” section, we compare CE-EM and a baseline methodology in their ability to fit partially observed disease data. Finally, in the “Conclusion” section, we discuss possible limitations to the scope of our work, as well as directions for further study.

## Background

In this section, we review compartmental SEIRD models, as well as CE-EM, an algorithm for fitting partially observed state-space models to data. We also discuss the literature addressing related problems.

### SEIRD models

SEIRD models are mathematical models of the spread of an infectious disease. Every individual in a population is in one of five states—they are either susceptible (S) to the disease, exposed (E) to the disease, infected (I) by the disease, or who have recovered (R) or died (D) from the disease^2^. In this context, we assume an exposed individual is ‘pre-symptomatic’, i.e., they can spread the disease but have not yet tested positive for it, while infected individuals are symptomatic and have tested positive for the disease. There are numerous extensions to this model^2,6,8^. For example, a SEIR model typically groups those who have recovered and died from the disease into a single state. SEIRD models may also differ in their modeling of reinfection, limited testing, or asymptomatic and quarantined individuals.

#### Compartmental models

In reality, each individual interacts with only a subset of individuals in the population. Thus the spread of the disease ought to be considered as propagating over a social interaction network of sparsely connected nodes. However, by introducing this fidelity into the model, the system’s state grows exponentially with the number of individuals, and the task of fitting the model to data becomes difficult. A compartmental SEIRD model ignores the network structure of a population by assuming that the population is *homogeneously mixed*. That is, we assume that every individual in the population is equally likely to interact with any other individual in the population on a given day. By making this assumption, we can dramatically simplify the state of the system. We need only track the number of individuals in each disease state, referred to as the *compartmental populations*, and not specific individuals.

We can model the dynamics of the state $x_t = [S_t, E_t, I_t, (RD)_t]^\top$ as the following deterministic system of differential equations:

1 $$\begin{aligned} \frac{d}{dt}\begin{bmatrix} S\\ E\\ I\\ RD \end{bmatrix} = \begin{bmatrix} -\left( \beta _E\frac{S_t E_t}{N} +\beta _I\frac{S_t I_t}{N}\right) \\ \beta _E\frac{S_t E_t}{N} +\beta _I\frac{S_t I_t}{N} - \gamma E \\ \gamma E - \lambda I \\ \lambda I \end{bmatrix}. \end{aligned}$$

Here, the parameters $\beta _E$ and $\beta _I$ (referred to as the *infection rates*) are interpreted as the average number of individuals that an exposed or infected individual comes in contact with per unit time, multiplied by the probability that the contact results in disease transmission, respectively. The parameter $\gamma$ specifies the average number of exposed individuals who transition to the infected state, per unit time, and $\lambda$ specifies the average number of infected individuals who recover or die, per unit time. We define infected individuals to be those who have *tested positive* for the disease, and thus $\gamma ^{-1}$ is the average amount of time it takes for someone who contracted the disease to test positive for it. The *recovered-deceased* (RD) compartment includes both individuals who have truly recovered from the disease and those who have died from it. Hence, $\lambda ^{-1}$ is the average amount of time it takes for someone to no longer be infectious after having tested positive, as a result of recovery or death. If we assume a fixed mortality rate $\mu$ for the disease, then we can compute the number of individuals who have died from the disease as $D_t = \mu (RD)_t$, and those who have recovered from it as $R_t = (1-\mu ) (RD)_t$.

The above framing describes a deterministic dynamical system. There are many methods for framing compartmental SEIRD systems using stochastic differential equations^9^, and one such formulation will be discussed in the “Methodology” section.

It is worth noting the various effects *not* modeled here. In this model, we do not account for individuals who never develop symptoms. Furthermore, this model assumes an individual’s infectiousness changes when they test positive—the truth of which may depend on the delays experienced with RT-PCR testing. SEIRD models can be extended to account for these effects, though such extensions may introduce more parameters and not be identifiable from the available data.

It would be straightforward to learn the parameters $\theta = [\beta _E, \beta _I, \gamma , \lambda , \mu ]$ from data if we could directly observe the compartmental populations. However, the available data is typically only of the aggregate transitions from the *E* compartment to the *I* compartment, when individuals test positive for the disease, and aggregate transitions from *I* to *R*, when individuals recover from or die due to the disease. As a result, estimation of $\theta$ must be performed under partial observability. Methods for doing so, including CE-EM, will be discussed next.

### Certainty-equivalent expectation maximization

Suppose we seek to find the parameters of the following state-space dynamical system:

2 $$\begin{aligned} \begin{aligned} x_{t+1}&= f_\theta (t, x_t) + w_t; \quad&w_t\sim p_w(\cdot ), \\ y_t&= h_\theta (t, x_t) + v_t; \quad&v_t\sim p_v(\cdot ). \end{aligned} \end{aligned}$$

Here, $x_t$ is the state of the system, $f_\theta (t,x_t)$ is a parameterized model of the dynamics, $w_t$ is an additive *process noise* term, and $p_w$ is the distribution from which $w_t$ is sampled. Furthermore, $y_t$ is an observation of $x_t$, $h_\theta (t,x_t)$ is a parameterized observation model, $v_t$ is the *observation noise* term, and $p_v$ is the distribution from which $v_t$ is sampled. In a *nonlinear Gaussian* system, $p_w$ and $p_v$ are multivariate Gaussian distributions.

Methods for parameter estimation typically attempt to quantify the likelihood $p(y_{1:T} \mid \theta )$ of a time-series of observations $y_{1:T}$ given some choice of parameters $\theta$. A set of methods called approximate Bayesian computing (ABC) use this estimate to characterize the Bayesian posterior $p(\theta \mid y_{1:T})$ using approximate methods such as Markov Chain Monte Carlo^10,11^. Maximum-likelihood (MLE) methods attempt to find the likelihood maximizing parameters $\theta _\text {ML} = \underset{\theta }{\arg \max }~p(y_{1:T} \mid \theta )$. Both methods rely on being able to estimate the data likelihood $p(y_{1:T}\mid \theta )$ with low-variance.

In order to estimate the data likelihood, we must marginalize over the unobserved states of dynamical system:

3 $$\begin{aligned} p(y_{1:T} \mid \theta ) = \int p(y_{1:T}, x_{1:T} \mid \theta )~dx_{1:T}. \end{aligned}$$

Many approaches to fitting SEIRD models to data assume known initial conditions $x_1$ when estimating $p(y_{1:T}\mid \theta )$^5,6,12^. Key drawbacks to such approaches are their sensitivity to the choice of $x_1$, as well as a degradation in their ability to find global optima if being fit to long time-series. Since computing this expectation is generally intractable without knowing initial conditions and for long time-series, many approaches instead find the posterior distribution over states, i.e. $p(x_{1:T} \mid y_{1:T}, \theta )$, and then approximate quantities of interest using Monte-Carlo samples^13^.

In a subset of MLE methods, the *smoothing distribution* $p(x_{1:T} \mid y_{1:T}, \theta$) is used to estimate the joint data log-likelihood:

4 $$\begin{aligned} Q(\theta , \theta ^k) = {\mathbf {E}}_{x_{1:T} \sim p(\cdot \mid y_{1:T}, \theta ^k)}\left[ \log p(y_{1:T}, x_{1:T} \mid \theta ) \right] . \end{aligned}$$

The function $Q(\theta , \theta ^k)$ is then maximized to yield $\theta ^{k+1}$, and this two-step procedure is repeated until convergence in an algorithm called Expectation-Maximization (EM)^14^. Such approaches overcome the need to precisely know $x_1$ and can straightforwardly handle long time-series^4^.

Various algorithms differ in how they compute the smoothing distribution. ParticleEM uses particle smoothing, an approach that uses sequential Monte-Carlo to approximately sample from the smoothing distribution^13,15^. Though this approach makes very few assumptions, it can require a prohibitive number of Monte-Carlo samples to yield sufficiently low variance estimates of $Q(\theta ,\theta ^k)$.

The Certainty-Equivalent EM (CE-EM) algorithm makes the approximation that $p(x_{1:T} \mid y_{1:T}, \theta )$ can be modeled by a Dirac-delta function located at the smoothing distribution’s mode^4^. In the case where $p_w(\cdot )$ and $p_v(\cdot )$ are Gaussian distributions, the assumptions made allow CE-EM to find an estimate for $\theta ^\text {ML}$ using block-coordinate ascent. By doing so, CE-EM finds solutions with significantly higher efficiency and lower variance than other estimation procedures^4^. However, by making this approximation, the algorithm is known to be biased in the presence of large process noise.

In this work, we propose using CE-EM to fit SEIRD models, including our proposed extension to available county-level data. As one might expect, such an approach should yield inaccurate results if the disease progression is highly stochastic, or poorly modeled by a compartmental model. However, we will show that even when fitting a single model to data from six counties with diverse epidemics, simulations from the model appear to reflect reality.

### Related work

Since the dawn of the COVID-19 pandemic, many efforts have been directed at forecasting its evolution, as well as fitting modified SEIR models to specific outbreaks^16,17^. Approaches vary in their characterization of the model, assumptions made, and their methodology for parameter estimation under partial observability. Korolev^5^ demonstrates issues with the identifiability of the SEIRD model and presents an estimation technique for the basic reproduction number $R_0$. They fit a standard SEIRD model to COVID-19 data, but assume known initial conditions to address partial observability—an assumption that can only be made in restricted settings. He et al.^6^ use particle swarm optimization to optimize the parameters of a SEIR model extended to account for hospitalized and quarantined individuals. Sun and Wang^7^ fit an extended SEIR model that accounts for a threshold in recovery rate, as well as asymptomatic patients. Both of these studies also address partial observability by assuming known initial conditions, but only fit to a single, short trajectory of data from the Hubei province and Heilongjiang province in China, respectively. Arik et al.^8^ introduce additional data sources such as mobility as covariates to expand the explanatory power of SEIR models, and specify a distribution over initial conditions to overcome partial observability. One of the more successful approaches to forecasting the early pandemic progression also fit SEIR models with a subset of variables that vary with time^12^. Their approach also assumes a known initial condition and finds parameters by minimizing simulation error. In contrast, our methodology does not need to assume knowledge of initial conditions, and instead infers unobserved states such that they maximize the likelihood of the data.

Recent works^18–20^ have also attempted to use neural networks to forecast the spread of COVID-19. While Wieczorek et al.^19^ and Melin et al.^20^ do not use neural-networks to model relationships within a SEIR model, Dandekar and Barbastathis^18^ use them to model a quarantine control function. Similar to other discussed methodologies, they assume initial conditions to overcome partial observability. Yang et al.^21^ fit both a SEIR model with additional compartments, as well as a black-box LSTM to a short time-series of COVID-19 infection data from Hubei, China, and compare their forecasting ability. Unlike our model and most other approaches, their SEIR model is not fit to the data by attempting to reproduce a time-series, but by linearizing the model around certain set points and assuming values for unknown quantities.

The methods mentioned above attempt to fit disease models that assume the population to be in one of many disease states, and specify differential equations that govern their rate-of-change. Once model parameters are estimated with a dataset, the methods perform forecasts by simulating the estimated disease states forward in time. Another approach commonly taken to forecasting the near-term future of a disease outbreak is to use statistical methods that directly attempt to predict a given quantity at some future point in time. For example, Castillo and Melin^22,23^ construct a fuzzy model that uses linear and nonlinear fractal dimensions of a time-series in order to make forecasts of future cases and deaths. Though such statistical models are useful for forecasting the evolution of the disease, they often lack interpretability and behavioral guarantees provided by disease models using differential equations to govern transitions between disease states.

To our knowledge, our work is the first to use a neural network to model the relationship between time, prevalence, and infection rate, the first to use Certainty-Equivalent EM for the purpose of parameter estimation under partial observability, and also the first to fit a single model to multiple time-series from across the United States.

## Methodology

This section describes data sources used in this work, the proposed modification to SEIRD models yielding R-SEIRD models, the formulation of R-SEIRD models as nonlinear Gaussian systems, and a procedure for fitting these models to available data.

### Data sources

In this work, the primary data source considered is the Novel Coronavirus (COVID-19) Cases Dataset, provided by JHU CSSE^24^, which provides a time-series of daily reported COVID-19 cases and deaths for every county in the United States.

### R-SEIRD models

As stated in the “Introduction” section, we propose to relax the assumption that the infection rate $\beta _E$ is constant with time. Instead, we model it to be a function of time and the *observed prevalence* of the disease. Specifically, we let:

5 $$\begin{aligned} \beta _E(t) = {\tilde{\beta }}_E\cdot \sigma \left( \text {NN}_{{\tilde{\theta }}}\left( t, \frac{I_t}{N_t}\right) \right) . \end{aligned}$$

Here, $N_t = S_t+E_t+I_t+(RD)_t$ is the *effective* population size at time *t*, a quantity that we allow to vary with time^25^ and be dynamically inferred during learning. Additionally, $I_t/N_t$ is the observed prevalence, $\text {NN}_{{\tilde{\theta }}}$ is a neural network mapping ${\mathbf {R}}^2\rightarrow {\mathbf {R}}$, $\sigma (\cdot )$ is the sigmoid function, and ${\tilde{\beta }}_E$ is a learned coefficient. For simplicity, we assume $\beta _I = 0$, which implies that the number of infections caused by an individual after they test positive for COVID-19 is negligible compared to the number of infections prior to them knowing they have the disease. Furthermore, since much is now known about the typical duration between exposure and symptom onset, and symptom onset and death, we rely on literature to provide estimates of $\gamma$ and $\lambda$^26,27^. Our methodology does, however, allow us to treat them as learned parameters. We treat the mortality rate $\mu$ as learned, and thus $\theta = [{\tilde{\beta }}_E, {\tilde{\theta }}, \mu ]$ are the learned parameters.

With the modification that $\beta _E$ depends on time, we allow the model to reflect changes in the infection rate that may depend on changing behaviors over time, such as climate, and the dominant strain of the virus. With the modification that it depends on prevalence, we allow for changes in infection rate in response to the level of infection in a county, as well as account for heterogeneous interaction patterns between geographies, that may affect the peak infection rates reached in those geographies.

### R-SEIRD as a nonlinear Gaussian system

In order to effectively learn the parameters of an R-SEIRD model using CE-EM, we represent it as a nonlinear Gaussian system. To do so, we must specify the state $x_t$, discrete-time dynamics function $f_\theta (t, x_t)$, observation $y_t$, and observation function $h_\theta (t, x_t)$.

Though the state of a SEIRD system is described by the number of individuals in each compartment, we note two facts about these quantities:

- The number of individuals in any compartment is a positive quantity, and,
- These quantities scale various orders of magnitude^24^.

For this reason, it is sensible to let the state $x_t$ of the system correspond to the logarithm of each compartment’s population as opposed to their absolute value. As a result, applying Gaussian process or observation noise to these quantities loosely corresponds to assuming that noise is proportional to the absolute value of the quantity it is applied to. Specifically, let the state of the system be $x_t = \log ~[S_t,E_t, I_t, (RD)_t]$, and the observation be $y_t = \log ~[\Delta C_t, \Delta D_t]$. Here, $\Delta C_t$ corresponds to the number of new confirmed cases on day *t* and tracks the total number of individuals that have transitioned from the *E* to the *I* compartment, and $\Delta D_t$ corresponds to the number of new deaths on day *t*.

The dynamics and observation models for the R-SEIRD model are then specified as follows:

6 $$\begin{aligned} \Delta \log \begin{bmatrix} S\\ E\\ I\\ RD \end{bmatrix}_t= & {} \int _t^{t+1}\begin{bmatrix} -\frac{\beta _{E}(\tau ) SE}{N} \\ \frac{\beta _{E}(\tau ) SE}{N} - \gamma E\\ \gamma E - \lambda I \\ \lambda I \\ \end{bmatrix}_\tau \circ \begin{bmatrix} 1/S\\ 1/E\\ 1/I\\ 1/RD \end{bmatrix}_\tau d\tau \nonumber \\&+ w_t;\quad w_t\sim \mathcal {N}(0,\Sigma _w), \end{aligned}$$

7 $$\log \left[ {\begin{array}{l} {\Delta C_{t} } \\ {\Delta D_{t} } \\ \end{array} } \right] = \log \left[ {\begin{array}{l} {\gamma E} \\ {\mu \lambda I} \\ \end{array} } \right] + v_{t} ;\quad v_{t} \sim{\mathcal{N}}(0,\Sigma _{v} ).{\text{ }}$$

where $\beta _{E}(t)$ is computed according to Equation 5. The process noise covariance $\Sigma _w$ and observation noise covariance $\Sigma _v$ are hyperparameters that can be chosen to be identity or diagonal matrices scaled by $\sigma _w^2$ and $\sigma _v^2$ respectively. Integration is performed using a Runge-Kutta method^28^.

Since the parameters ${\tilde{\beta }}_E$ and $\mu$ are positive, we optimize their logarithms as opposed to their absolute values. Framed as a nonlinear Gaussian system, the R-SEIRD model can straightforwardly fit a batch of observation time-series from multiple counties simultaneously by using CE-EM^4^. To improve fit reliability and eliminate periodic drops from weekends, we apply a 7-day moving average filter to daily case and death data before fitting to them.

In our experiments, we fit the R-SEIRD model to data from six counties in the United States exhibiting diversity in how COVID-19 has spread, and show that it is capable of expressing this diversity.

## Experiments

In this section, we first perform an experiment to fit SEIRD and R-SEIRD using CE-EM to COVID-19 data from counties across the United States. In a second experiment, we generate simulated data to compare the use of CE-EM and a baseline methodology in their ability to fit partially observed disease data.

### Fitting COVID-19 epidemics across The United States

Our experiment aims to achieve the following goals:

1. Fit a single R-SEIRD model to data from a variety of counties across the United States,
2. Show that the learned model, when simulated with realistic initial conditions, can reproduce observed behavior, and,
3. Justify the modifications to the SEIRD model by showing that the mean squared error (MSE) of simulations under the R-SEIRD model is much lower than that of a fit SEIRD model.

To achieve these goals, we fit models to data from three counties in the northeast of the United States (Middlesex, MA, Kings, NY, and Fairfield, CT), as well as three counties across the United States that have had diverse epidemics (Los Angeles, CA, Miami-Dade, FL, and Cook, IL). We consider data from February 22, 2020, to September 27, 2020, because during this period, the counties in the northeast exhibit single, clear peaks in infection, though the remaining counties exhibit multiple peaks. The remaining worst-hit counties in each of the United States compose a test-set to evaluate the model.

We fit both an R-SEIRD model and a standard SEIRD model (where we learn just the constant parameters $\beta _E$ and $\mu$) to data from these six counties, and then attempt to see if the learned models can reproduce the behavior of all six counties, as well as of counties not trained on, from appropriate initial conditions. We visually compare simulations on select counties to show that deterministic simulations of the R-SEIRD system can express multiple peaks while those of a SEIRD system cannot. We also visualize the learned relationship between time, prevalence, and infection rate, to provide an intuition for how the model can express such behavior. We then compare the MSE of simulations from both learned models on the worst-hit county of each of the United States (not in the training set), showing that simulation error is much lower when using the R-SEIRD model. The codebase for running these experiments can be found at https://github.com/sisl/rseird.

### Experimental setup

Here we detail the hyperparameters used when training the R-SEIRD and SEIRD models, the methodology for selecting appropriate initial conditions for states when evaluating the models, as well as metrics for evaluation.

#### Hyperparameters

When learning the R-SEIRD model or SEIRD model, we specify the values of $\gamma$ and $\lambda$. The literature suggests that the median time from exposure to developing symptoms is five days^27^ and that the median time between symptom onset and recovery is 21 days^26^. As a result, we let $\gamma = 1/5$ and $\lambda =1/21$. We let $\sigma _w = \sigma _v/10$, assuming that corruptions to observations are an order of magnitude noisier than corruptions to the process. Furthermore, we let the observation noise on deaths be double that on cases, since reporting is typically noisier when the number of deaths is small. The neural network used in the R-SEIRD model has three hidden layers with 32 hidden units each and tanh activation functions. Both models are optimized in the CE-EM learning step using an Adam optimizer with a learning rate of $5\times 10^{-4}$, which is harmonically decayed over time, and CE-EM trust-region parameters of $\rho _x=0.5$ and $\rho _\theta =0.01$^4^. We additionally smooth the observed data using a 7-day moving average filter to smooth high-frequency noise, including the expected drop in cases over weekends.

#### Initial condition selection

When evaluating learned models, partial observability makes it such that we do not know the initial conditions (i.e., the number of individuals in each disease compartment) to simulate the models from. Related work often assumes the population is almost completely unexposed at $t=1$, and assumes the susceptible population is a community’s true population^6^. However, as stated in the “Methodology” section, we allow the population size to be determined by the sum of the compartmental population, and therefore be a free parameter. Doing so is necessary because, for trajectories in our dataset, it is unreasonable to assume the population is initially unexposed.

For these reasons, we optimize for the initial conditions from which deterministically simulated trajectories minimize the MSE of the observed number of daily cases. We optimize the initial conditions by using the Cross-Entropy Method^29^, selecting the top 100 candidates at each epoch, perturbing each candidate to generate 1000 candidates for the next epoch, and running the optimizer for 10 epochs.

#### Evaluation metrics

When selecting initial conditions, we measure the MSE between the log of the daily cases in simulation (i.e., $\log (\gamma E_t)$), and reality (i.e., $\log \Delta C_t$), and use the distribution over this metric to compare the R-SEIRD and standard SEIRD models. We compare histograms of these errors on data from counties not in the training set.

#### Results

**Figure 1:**
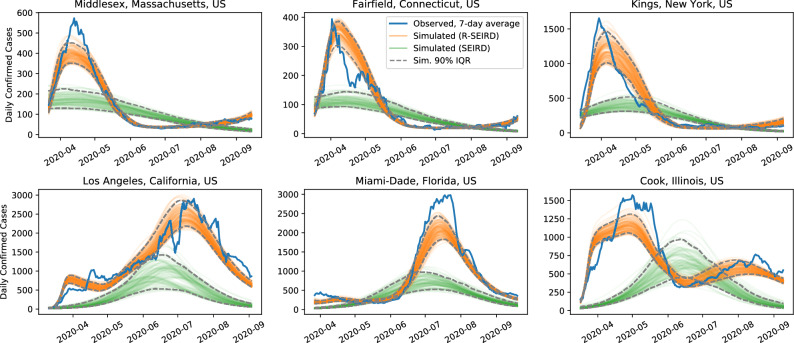
Simulations of the learned R-SEIRD and learned SEIRD systems on the six counties the models are trained on. The trajectory in blue is the 7-day moving average of observed daily confirm cases. These results demonstrate the expressiveness of our model. In particular, our model (orange) is able to track observed disease progression (blue) much closer than the canonical SEIRD model (green). In addition, it is able to identify the observed multiple peak behavior for which SEIR is not designed to capture. These results are consistent across diverse geographic areas of US.

In Fig. 1, we see the simulated number of cases from optimized initial conditions for the six counties on which the R-SEIRD model was trained. As is clearly seen, the model can express both a single clear peak present in the northeastern counties and the multiple irregular peaks present in the other counties. Since the simulations are deterministic, they are smooth and only approximately reflect the trends in the trajectories they are supposed to match.

In contrast, and consistent with expectation, a SEIRD model, even when fit to the same data and simulated from optimized initial conditions, cannot reflect the multiple peaks in the training data. Furthermore, in an attempt to fit the multi-peak behavior in three counties, the fit is severely compromised in counties with a single, clear peak.

**Figure 2:**
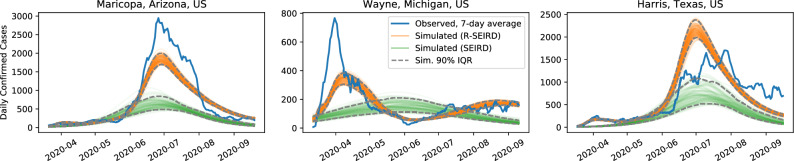
Simulations of the learned R-SEIRD and learned SEIRD systems on the three counties the models are not trained on. The trajectory in blue is the 7-day moving average of observed daily confirm cases. These results demonstrate the generalizability of our model against unseen data. We show representative results across different geographic regions of how our model can more accurately predict the different signatures of disease progression. Note that the SEIRD model consistently under-estimates the prevalence of disease spread, which has significant implications for policy-making decisions on the severity of intervention.

In Fig. 2, we compare the learned R-SEIRD and SEIRD models on three hard-hit counties not in the training set. Again, the SEIRD model cannot express more than a single peak, and thus cannot capture the behavior reflected in reality. Despite not being trained on data from these counties, the R-SEIRD model is, however, able to reflect the double-peaks seen in the data, which vary in onset and magnitude.

**Figure 3:**
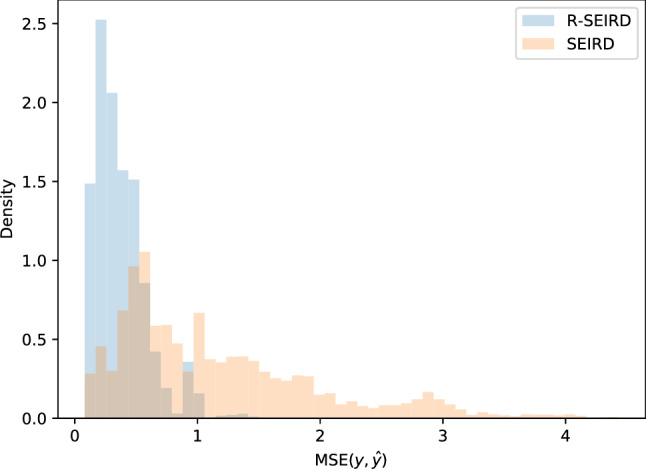
Comparison of prediction errors between R-SEIRD and SEIRD for the epidemic in the worst-hit county of each of the United States. We observe the errors of our model are substantially smaller than the canonical SEIRD model.

We quantify the difference in simulation quality by measuring the MSE between the simulations (from optimized initial conditions) with the observed trajectories. In Fig. 3, we show a histogram of these errors for simulations on the worst-hit county of each of the United States (not in the training set). Consistent with the improvement visible in Fig. 2, we see that the MSE of simulations by the learned R-SEIRD model is significantly lower than that of the SEIRD model.

**Figure 4:**
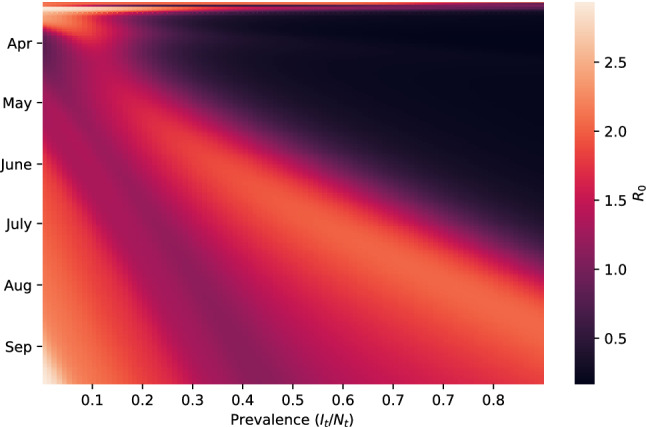
The learned mapping between time, prevalence, and the infection rate. The infection rate is represented by the $R_0$ value and represented by the intensity of the color, with higher values reflecting more a infectious disease. This analysis aids to the interpretability of the deep learning model. It can lead to meaningful insights about potential intervention strategies.

To gain an intuition for how the R-SEIRD model is able to express multiple irregular peaks, we visualize the learned mapping between time, the prevalence of the disease ($I_t / N_t$) and the basic reproduction number $R_0 = \beta _E(t, I_t/N_t)/\gamma$, which indicates a growing epidemic for values greater than 1. In Fig. 4, we see the learned mapping, which suggests that $\beta _E$ decreases initially as prevalence increases, up until a point, after which it begins to surge again before decreasing. This behavior would be consistent with a two-tiered response, in which weak restrictions were adopted until they lost effect, and then stronger restrictions were imposed. Over time, we see that $\beta _E$ begins to require larger prevalence levels to decay and bring $R_0$ below one, suggesting that communities are growing more reluctant to adopt the weak restrictions when infection rates are low.

### Inferring versus assuming initial conditions

Our second experiment aims to compare CE-EM with a baseline methodology that assumes known initial conditions, for fitting compartmental models to partially observed data. Specifically, we aim to show that:

1. CE-EM more accurately estimates the values of unobserved quantities, such as the number of exposed and infected individuals at a given time, and,
2. Estimating the effective population size with CE-EM leads to better fits to the data than by assuming its value.

To do so, we simulate a disease spreading through a population of sparsely connected individuals, but only partially observe the aggregated disease states of the population. We then attempt to fit a compartmental SEIRD model to the observed data using both CE-EM and a baseline methodology. We demonstrate that CE-EM is better at recovering unobserved states than the baseline methodology, and produces a better quality fit to the data.

#### Baseline methodology

We baseline against a common approach^5,6,8,12^ that assumes the initial conditions of unobserved state-variables are known, which we refer to as the Fixed Initial Condition (FIC) baseline. Specifically, we let ${\tilde{x}}_1$ be the assumed initial condition, and let $\theta$ be the parameters of a deterministic compartmental disease model $x_{t+1} = f_\theta (x_t), y_t = g_\theta (x_t)$. We can forward-simulate the initial conditions according to the disease model to yield a state-trajectory ${\tilde{x}}^\theta _{1:T}$, and observation-trajectory ${\tilde{y}}^\theta _{1:T} = g_\theta ({\tilde{x}}^\theta _{1:T})$. We can then define a loss:

8 $$\begin{aligned} {\mathcal {L}}(\theta ) = \Vert {\tilde{y}}^\theta _{1:T} - y_{1:T} \Vert ^2_2 \end{aligned}$$

and minimize the loss with respect to $\theta$.

To overestimate the performance of the FIC baseline methodology, we provide the baseline methodology with the exact initial condition of the unobserved disease state. Furthermore, ${\mathcal {L}}(\theta )$ is nonconvex, and thus we optimize the objective using Differential Evolution to explore more of the space for a global optimum.. It is possible to use a global optimizer on this problem because the dimensionality of $\theta$ is low.

#### Simulated data

To generate simulated diseases data, we consider a population in which individuals are sparsely connected to one another. We generate this graph by sampling the ‘locations’ of 10,000 individuals as random points on a unit square. We then assign an edge between two individuals *i* and *j* with a probability that exponentially decays with the distance between them. In this experiment, individuals have an average of 6.126 connections between them.

Individuals in the population can each be in one of five states: susceptible (S), exposed (E), infected (I), recovered (R), or dead (D). At a given instant in time, a susceptible individual becomes exposed with probability:

9 $$\begin{aligned} p^{S\rightarrow E}_i = 1 - (1-\beta _E)^{\sum _{j\in {\mathcal {N}}(i)}E(j)}, \end{aligned}$$

where ${\mathcal {N}}(i)$ is the set of individuals that individual *i* is connected to, and *E*(*j*) is 1 if individual *j* is exposed, and otherwise 0. Furthermore, every exposed individual transitions to the infected state with probability $\gamma$, and every infected individual to the recovered state with probability $\lambda (1-\mu )$, and to the dead state with probability $\lambda \mu$. We simulate an outbreak initialized with 1% of randomly chosen individuals in the exposed state, and the remainder in the susceptible state. We let $\beta _E = 0.06, \gamma =1/5, \lambda =1/21$ and $\mu =0.1$, and we simulate 175 days of the outbreak. At every time-step, we observe only the number of individuals who have transitioned from the *E* to *I* compartment, and the number who have transitioned from the *I* to *D* compartment.

**Figure 5:**
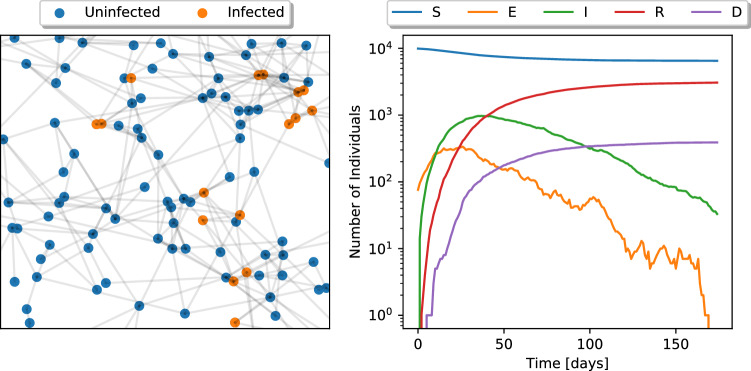
(Left) Depiction of the graph connecting the population, and the individuals infected or left uninfected over the course of the simulation. (Right) Aggregated numbers of individuals in each disease state over the course of the simulation. We see that due to the network’s sparsity, the disease only propagates through a subset of the population, affecting just $\sim 30\%$ of individuals.

In Fig. 5, we see both a depiction of the graph connecting the population, and the aggregated number of individuals in each disease state over the course of the simulation. Since the population is sparsely connected (which is a realistic property of observed interaction networks), only $\sim 30\%$ is ever infected with the disease.

#### Results

**Figure 6:**
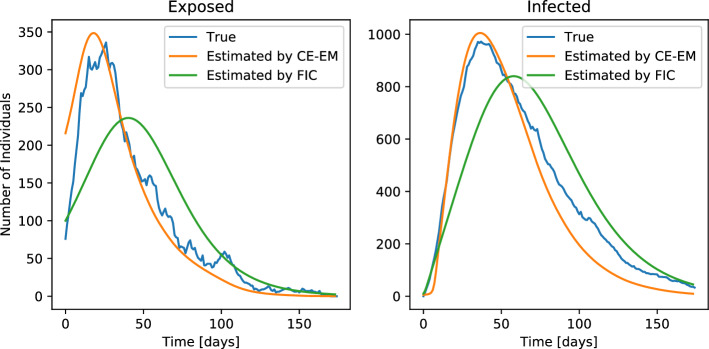
Estimation of unobserved quantities by CE-EM and FIC baseline methods when fitting a SEIRD model to simulated data. (Left) Estimation of the number of exposed individuals. (Right) Estimation of the number of infected individuals. The estimates made by CE-EM align almost perfectly with ground truth in terms of peak intensity, peak height, and curve shape, while those of the FIC baseline do not.

In Fig. 6, we show the aggregated number of exposed and infected individuals over the course of the simulation. These two quantities are not directly observed during training, and are thus estimated by the compared algorithms. Overlaid on these trajectories are the trajectories estimated by CE-EM and the FIC baseline methodology. We see that the trajectories estimated by CE-EM almost perfectly align with the ground truth in their shape, peak location, and peak magnitude. Those estimated by the FIC baseline methodology, however, align poorly with the ground truth.

We quantitatively compare the mean-square-error (MSE) of predicted observations to those observed during the simulation. We predict observations using the models learned by each method and the states estimated by each learning methodology. We find that the MSE of predicted observations is 0.046 when using CE-EM and 0.241 when using the FIC baseline methodology.

The qualitative and quantitative comparisons between these methods show that CE-EM more accurately estimates the values of unobserved quantities, such as the number of exposed and infected individuals at a given time. They also show that estimating the effective population size instead of assuming its value, CE-EM leads to better fits to the data than by using the FIC baseline methodology.

## Conclusion

In this work, we proposed an extension to the compartmental SEIRD model that relaxes the assumptions of static model parameters and non-reactivity to disease prevalence called the R-SEIRD model. We did so by training a neural network to map the time and the prevalence of the disease to the infection rate. In order to fit available data, we employed Certainty-Equivalent Expectation-Maximization (CE-EM), which is a technique suited to fitting nonlinear Gaussian state-space models to data without direct observation of the system’s state variables, and does not assume knowledge of the system’s initial conditions. We provided a methodology for framing the R-SEIRD model as a nonlinear Gaussian system, and for fitting it to available data on daily confirmed cases and deaths.

Our experiments fit both the R-SEIRD and standard compartmental SEIRD models to data from six counties across the United States. We showed that the R-SEIRD model learned is capable of expressing the range of multi-peak behavior exhibited not only in the training data, but also on counties not trained on. We showed quantitatively that the simulation error when trying to reproduce the behavior of the worst-hit counties in the United States is much lower when using the R-SEIRD model compared to the standard SEIRD model. We further justify the use of CE-EM as a methodology for fitting compartmental disease models in partially observed settings by showing it achieves better fits to simulated data than baseline methodologies that assume initial conditions are known.

This work showed that by allowing the infection rate to be time-varying and reactive, that the much more complex behaviors exhibited by the epidemic across the United States can be recovered. However, we do not suggest that the R-SEIRD model definitively explains why a given outbreak progressed the way it did, but instead proposes a hypothesis that is consistent with observations. To come closer to a definitive explanation, we must depart from another assumption made by both standard compartmental SEIRD and R-SEIRD models—that of *homogeneous mixing*. By explicitly modeling the observed heterogeneous social interaction patterns in a county’s population, we gain the capacity to account for super-spreading individuals, as well as non-uniform population densities.

In follow-on work, we propose a SEIRD model that accounts for the network structure of a community and an estimation procedure for fitting it to data. With such a model, we gain another explanatory tool to analyze the diversity in outbreaks and a model that can be used to evaluate localized containment strategies at a community level. We intend to expand on the ideas behind CE-EM by using probabilistic programming to perform inference in network-based epidemiological simulations, thereby explicitly modeling the dependence on known interaction patterns. Additionally, we intend to apply these methodologies to outbreaks in other parts of the world, which might differ substantially from the United States in climate, cultural norms, disease variants, and containment measures. While R-SEIRD is flexible enough to be directly applied to aggregated data from any locality, certain regions may warrant the relaxing of assumptions beyond that which is done in this work. In a future study, we seek to understand which regions of the world sufficiently share characteristics with the United States, so that we may use a single model to explain behavior across regions.

## Data Availability

The datasets generated and/or analyzed during the current study are available in the COVID-19 Data Repository by the Center for Systems Science and Engineering (CSSE) at Johns Hopkins University (https://github.com/CSSEGISandData/COVID-19)^24^.
